# Flunarizine-Loaded Hydrogels: A Novel Formulation and Physicochemical Characterization

**DOI:** 10.3390/polym18091014

**Published:** 2026-04-22

**Authors:** Camelia Daniela Ionaș, Dorinel Okolišan, Camelia Epuran, Ion Frățilescu, Gabriela Vlase, Alexandru Pahomi, Raul Ștefan-Pantiș, Mihaela Maria Budiul, Mădălina Grădinaru, Titus Vlase

**Affiliations:** 1 Research Centre for Thermal Analysis in Environmental Problems/ICAM-Advanced Environmental Research Institute, West University of Timisoara, Oituz Street 4, 300233 Timisoara, Romania; camelia.ionas@e-uvt.ro (C.D.I.); dorinel.okolisan11@e-uvt.ro (D.O.); camelia.epuran@e-uvt.ro (C.E.); ion.fratilescu@e-uvt.ro (I.F.); alexandru.pahomi@e-uvt.ro (A.P.); raul.stefan00@e-uvt.ro (R.Ș.-P.); madalina.mateescu@e-uvt.ro (M.G.); titus.vlase@e-uvt.ro (T.V.); 2 Doctoral School of Exact Sciences and Natural Sciences, West University of Timișoara, Pestalozzi Street 16, 300115 Timisoara, Romania

**Keywords:** flunarizine, hydrogels, FTIR, thermal analysis, HPLC

## Abstract

Flunarizine is a calcium channel blocker widely used in neurological disorders; however, its low aqueous solubility may influence formulation stability and drug dispersion in polymer-based systems. The present study aimed to evaluate the compatibility of flunarizine with selected excipients and to investigate its incorporation into polymeric hydrogel matrices. Binary mixtures of flunarizine with excipients such as hydroxypropyl-β-cyclodextrin, polyethylene glycol (PEG 6000), Tween 20, gelatin, and citric acid were prepared and characterized using Fourier-transform infrared spectroscopy (FTIR), thermogravimetric analysis (TG/DTG), and high-performance liquid chromatography (HPLC). The FTIR spectra of the analyzed samples do not reveal the appearance of new absorption bands that may indicate chemical interactions; instead, minor spectral variations were observed due to weak intermolecular interactions within the polymer network. Thermal analysis revealed decomposition patterns consistent with those of the individual components, suggesting the absence of significant incompatibilities. A validated RP-HPLC method enabled sensitive and reliable quantification of flunarizine in the analyzed systems, with a limit of detection (LOD) of 0.05 µg/mL and a limit of quantitation (LOQ) of 0.16 µg/mL. Accuracy testing showed average recovery rates of 100% across 80–120% spiking levels. Overall, the results support the compatibility of flunarizine with the investigated excipients and the suitability of the studied hydrogels as potential drug delivery matrices.

## 1. Introduction

Polymeric materials play an essential role in modern pharmaceutical formulations, particularly in the design of advanced drug delivery systems. Hydrogels, in particular, have attracted considerable attention due to their three-dimensional polymeric networks capable of retaining large amounts of water while maintaining structural integrity. These systems offer several advantages, including improved drug dispersion, controlled release profiles, and enhanced stability of active pharmaceutical ingredients. As a result, polymer-based hydrogels have been widely explored as carriers for a variety of therapeutic compounds, especially for drugs with limited aqueous solubility or complex physicochemical properties. Understanding the interactions between drugs and polymeric matrices is therefore crucial for the rational development of stable and effective pharmaceutical formulations [1,2].

**Figure 1:**
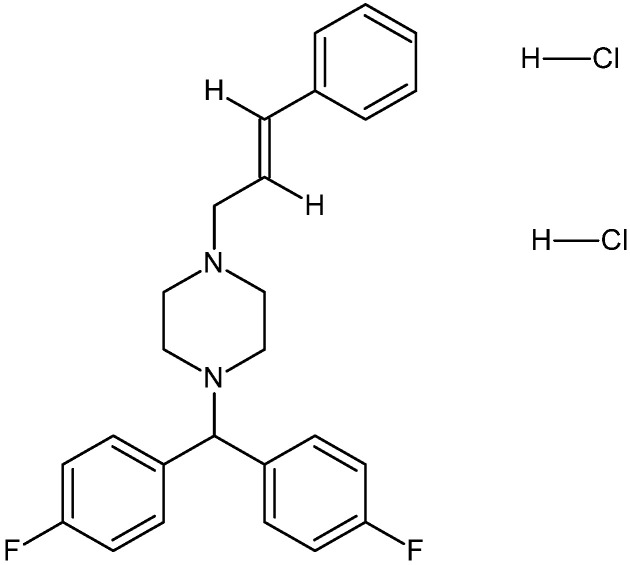
Chemical structure of flunarizine dihydrochloride (adapted from PubChem database) [8].

Flunarizine, or flunarizine dihydrochloride (Figure 1), C_26_H_26_F_2_N_2_·2HCl, is a non-selective calcium channel blocker commonly used in the prophylactic treatment of migraine and vestibular disorders. Chemically, flunarizine is a diphenylmethylpiperazine derivative whose aromatic rings, tertiary amine groups and fluorinated substituents in the flunarizine molecule may facilitate weak intermolecular interactions such as hydrogen bonding or van der Waals interactions with polymeric excipients, which may influence drug stability and dispersion within polymer-based systems. Due to its pronounced lipophilicity and low aqueous solubility, flunarizine presents formulation challenges that may affect its stability, dispersion, and bioavailability within pharmaceutical systems [3,4,5]. These structural features also suggest a potential for interaction with polymeric carriers such as cyclodextrins or polyethylene glycols, which are frequently used to improve the solubility and stability of poorly soluble drugs [6,7].

During the preformulation stage of drug development, the compatibility between active pharmaceutical ingredients and excipients must be carefully evaluated, as drug–excipient interactions may significantly influence the stability and performance of pharmaceutical formulations. Such interactions can involve hydrogen bonding, electrostatic interactions, or inclusion complex formation, particularly when polymeric carriers or cyclodextrin derivatives are involved. Analytical techniques such as Fourier-transform infrared spectroscopy (FTIR), thermogravimetric analysis (TG/DTG), and high-performance liquid chromatography (HPLC) are commonly employed to investigate these interactions and to assess the physicochemical behavior of drug–excipient systems [6,9,10].

Several studies have explored the use of polymeric carriers and cyclodextrin derivatives to improve the physicochemical properties and formulation performance of poorly soluble drugs [2,7]. Cyclodextrins, polyethylene glycols, and other polymeric excipients have been widely investigated for their ability to enhance drug solubility, stability, and dispersion through weak intermolecular interactions or inclusion complex formation [7]. However, despite the extensive use of these excipients in pharmaceutical systems, studies specifically addressing the compatibility and physicochemical behavior of flunarizine in polymer-based matrices remain limited, particularly in the context of hydrogel formulations.

Therefore, the aim of the present study was to investigate the compatibility of flunarizine dihydrochloride with selected pharmaceutical excipients and to evaluate its incorporation into polymer-based hydrogel systems. Binary mixtures containing flunarizine dihydrochloride and different excipients, as well as the developed hydrogel formulations, were characterized using FTIR spectroscopy and thermogravimetric analysis (TG/DTG) as a preliminary assessment of possible interactions and thermal stability. The hydrogel formulations were further evaluated using high-performance liquid chromatography (HPLC).

## 2. Materials and Methods

Flunarizine dihydrochloride was used as the active pharmaceutical ingredient, produced by Mikromol (LOT: G1089929; LGC GmbH, Luckenwalde, Germany). The excipients employed in this study were hydroxypropyl-β-cyclodextrin (HP-β-CD; CycloLab R&D Ltd., Budapest, Hungary), polyethylene glycol by Sigma-Aldrich (PEG 6000; Merck KGaA, Darmstadt, Germany), Tween 20 (VWR International S.A.S, Fontenay-sous-Bois, France), gelatin (Merck KGaA, Darmstadt, Germany), glucose (Lach-Ner S.R.O., Neratovice, Czech Republic), sorbitol by Sigma-Aldrich (Merck KGaA, Darmstadt, Germany), and citric acid (Merck KGaA, Darmstadt, Germany). All chemicals and reagents used were of analytical grade and were used without further purification. Ultrapure water was used in the preparation of all solutions.

- Binary mixtures

Binary mixtures of flunarizine dihydrochloride (FLU) with selected excipients were prepared in a mass ratio of 1:1 (*w*/*w*) in order to evaluate possible drug–excipient interactions. The components were accurately weighed and subsequently mixed with a pestle in a mortar for 5 min to ensure homogeneity of the resulting binary mixture. The prepared mixtures were stored in sealed containers at room temperature until further analysis.

- Hydrogel formulations

Hydrogel formulations were prepared using a gelatin-based matrix.

For the PEG-based formulation, flunarizine dihydrochloride (0.05 g) was incorporated as a solid dispersion with polyethylene glycol 6000 (0.20 g), corresponding to a 1:4 mass ratio. Tween 20 (0.05 g, ~0.2% *w*/*w*) was added as a surfactant. The hydrogel matrix was prepared using gelatin (2.50 g), glucose (6.25 g), sorbitol (2.50 g), and citric acid (0.10 g), dissolved in purified water (13.85 g) under continuous stirring and controlled heating to obtain a homogeneous system.

For the HP-β-cyclodextrin-based formulation, flunarizine dihydrochloride (0.05 g) was incorporated with hydroxypropyl-β-cyclodextrin (0.25 g), corresponding to an approximate 1:5 mass ratio. The hydrogel matrix consisted of gelatin (2.50 g), glucose (6.25 g), sorbitol (2.50 g), and citric acid (0.10 g), dissolved in purified water (13.75 g) under continuous stirring and mild heating to obtain homogeneous systems.

In addition, corresponding hydrogels without flunarizine dihydrochloride were prepared using the same compositions and procedures for each gel base.

The obtained hydrogels were allowed to equilibrate at room temperature prior to further characterization.

- FTIR analysis

Fourier-transform infrared spectroscopy was used to investigate the obtained hydrogels and to evaluate the compatibility between flunarizine dihydrochloride and the selected excipients. FTIR spectra were recorded using a Shimadzu IRTracer-ATR spectrometer (Shimadzu Corporation, Tokyo, Japan). Spectra were collected over the range of 4000–400 cm^−1^, with a spectral resolution of 4 cm^−1^ and 20 scans per sample. The obtained spectra were examined for shifts, disappearance, or appearance of characteristic absorption bands, indicating possible drug–excipient interactions.

- Thermogravimetric analysis

Thermal behavior of the investigated samples, including hydrogel formulations and binary mixtures of flunarizine dihydrochloride with selected excipients, was evaluated using thermogravimetric analysis. TG and DTG curves were recorded using a Mettler TOLEDO TGA/DSC3+ thermogravimetric analyzer (Mettler-Toledo LLC, Columbus, OH, USA). Approximately 5 mg of each sample was heated from 25 °C to 500 °C at a heating rate of 10 °C/min, under a synthetic air atmosphere (20 mL min^−1^).

- RP-HPLC analysis

In this study, chromatographic analysis was performed using an Agilent 1100 Series system(Agilent, Waldbronn, Germany) equipped with a G1379A vacuum degasser, a G1311A quaternary pump, a G1311A autosampler, a G1316A column thermostat, and a G1365B multiple wavelength detector (MWD).

All solvents and reagents employed in this study were of analytical grade. The active pharmaceutical ingredient (API) standard was procured from LGC. Ultrapure water was freshly prepared using a Merck Milli-Q system. Acetonitrile of gradient-grade quality was obtained from Merck. Ammonium formate was sourced from Sigma-Aldrich, while formic acid was purchased from Honeywell Riedel-de Haën.

- Preparation of mobile phases

Mobile phase A—weigh 2.84 ± 0.06 g of ammonium formate and transfer it into a clean beaker; add 800 mL of ultrapure water; stir the solution using a magnetic hot plate stirrer for 10 min at 800 rpm. Transfer the solution to a 1 L volumetric flask, washing the beaker 3 times with 20 mL of UP water; add 4 mL of formic acid; then bring the solution to volume using ultrapure water. Filter the solution through a 0.45 µm nylon membrane, suitable for HPLC. Transfer the filtered solution into an HPLC solvent bottle and degas it using an ultrasonic bath at room temperature for 30 min.

Mobile phase B—pure acetonitrile—was poured directly into an HPLC solvent bottle and degassed by sonication at room temperature for 20 min.

- Preparation of the standard solution

The standard solution was prepared by weighing 4.98 mg of flunarizine standard in a 10 mL volumetric flask on an analytical scale. The standard was dissolved in 8 mL of methanol by sonication at room temperature for 5 min, after which the volume was adjusted with methanol to the mark on the volumetric flask. The same solution was used for system suitability testing. The same solution was used for system suitability testing (SST).

- Preparation of sample solution

One hydrogel unit was accurately weighed, and its mass was recorded; then it was cut into small pieces using a clean scalpel. The pieces were quantitatively transferred into a 10 mL volumetric flask, and approximately 5 mL of methanol was added to extract the flunarizine. The sample was sonicated for 1 h at 40 °C to ensure complete extraction of the active ingredient. After sonication, the solution was allowed to reach room temperature. The solution was transferred to a clean beaker, and a 2 mL portion was filtered through a syringe fitted with a PES membrane filter (0.45 µm) directly into a clean vial, discarding the first few drops of filtrate.

## 3. Results and Discussion

### 3.1. FTIR

**Figure 2:**
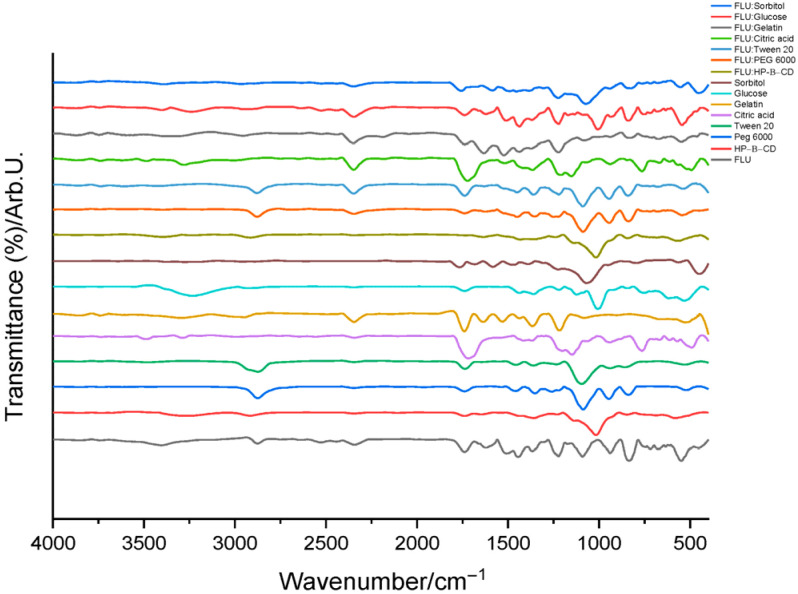
FTIR spectra of the active ingredient, the individual compounds of the hydrogels and binary mixtures.

To investigate potential interactions between flunarizine dihydrochloride (FLU) and the selected excipients, FTIR analysis was performed on binary mixtures as a preliminary compatibility assessment, in line with previously reported approaches, followed by the evaluation of the final hydrogel formulations. The spectra were recorded for the individual components, the binary mixtures of FLU with the excipients, and the final hydrogel systems, in the presence and absence of the active substance. The spectra of the active ingredient, individual components and binary mixtures are illustrated in Figure 2.

The FTIR spectrum of pure FLU shows characteristic absorption bands corresponding to its molecular structure, as previously described in the literature. The stretching vibrations of aromatic C=C bonds are observed in the region of approximately 1600–1500 cm^−1^, while additional bands associated with vibrations of the aromatic ring appear between 1450 and 1400 cm^−1^. A broad band around 3500 cm^−1^ is attributed to O–H stretching vibrations associated with absorbed moisture, reflecting the pronounced hygroscopic nature of flunarizine dihydrochloride [11]. Bands around 2900 cm^−1^ are assigned to C–H stretching vibrations, including contributions from the ethenic linkage connecting the aromatic and piperazine rings. The presence of C–F bonds is indicated by an intense absorption band around 500 cm^−1^. Furthermore, the band at approximately 850 cm^−1^ suggests a 1,4-disubstituted aromatic ring, while the signals in the 600–750 cm^−1^ region, appearing as two peaks of similar intensity, are characteristic of a monosubstituted aromatic ring associated with the ethenic fragment. Signals in the 1200–1000 cm^−1^ range are attributed to C–N stretching vibrations and contributions from the fingerprint region typical of substituted aromatic compounds [10,12].

The excipients used in the formulations exhibit characteristic bands corresponding to their specific chemical functional groups, in agreement with previously reported data. HP-β-cyclodextrin shows a broad O–H stretching band in the 3400–3200 cm^−1^ region, associated with the extensive hydrogen-bonding network, together with a band around 1650 cm^−1^ attributed to absorbed water within the oligosaccharide structure, as well as bands in the 1150–1000 cm^−1^ range, typical for C–O and C–O–C vibrations in cyclodextrin derivatives [7]. These bands fall within the characteristic region of polysaccharides (900–1200 cm^−1^), where structural features of carbohydrate-based systems can be identified. PEG 6000 presents intense bands around 1100 cm^−1^ attributed to C–O–C stretching vibrations of the polyether chain. Tween 20 exhibits the characteristic C=O stretching band of the ester group at approximately 1735 cm^−1^, accompanied by C–O stretching vibrations in the 1000–1100 cm^−1^ region. A strong band around 2900 cm^−1^, assigned to C–H stretching vibrations of aliphatic chains, is observed for both PEG 6000 and Tween 20, reflecting the presence of aliphatic (propyl) segments in their structures [13,14].

Other excipients used, such as gelatin, glucose, sorbitol, and citric acid, display characteristic vibrations of hydroxyl and carbonyl groups typical of polyhydroxylated compounds and organic acids, in agreement with general FTIR assignments reported in the literature [13,14].

HP-β-cyclodextrin shows a broad O–H stretching band in the 3400–3200 cm^−1^ region, associated with the extensive hydrogen-bonding network, together with a band around 1650 cm^−1^ attributed to absorbed water within the oligosaccharide structure, as well as bands in the 1150–1000 cm^−1^ range, typical for C–O and C–O–C vibrations in cyclodextrin derivatives [7]. These bands fall within the characteristic region of polysaccharides (900–1200 cm^−1^), where structural features of carbohydrate-based systems can be identified. PEG 6000 presents intense bands around 1100 cm^−1^ attributed to C–O–C stretching vibrations of the polyether chain.

**Figure 3:**
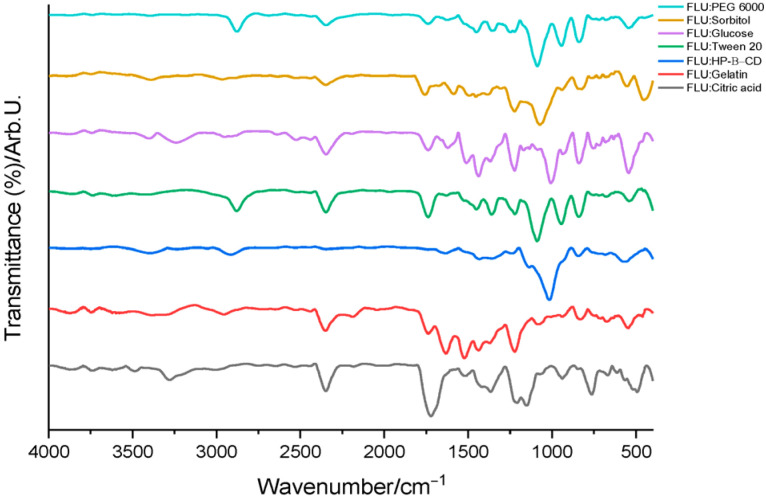
FTIR spectra of the binary mixtures.

The FTIR spectra of the binary mixtures of FLU with the studied excipients, presented in Figure 3, largely correspond to the superposition of the characteristic bands of the individual components. No new bands or significant disappearance of the characteristic bands of flunarizine were observed, indicating that the main structural features of the active substance are preserved in the mixtures. In some cases, minor variations in band intensity or slight shifts in band position were detected, particularly in the hydrogen-bonding region (3400–3200 cm^−1^) and in the aromatic vibration region (1600–1500 cm^−1^). These variations are consistent with weak intermolecular interactions, such as hydrogen bonding or dipole–dipole interactions, and do not suggest the formation of new chemical bonds [10,14]. Similar observations have been reported in studies on flunarizine solid dispersions with polyvinylpyrrolidone, where FTIR analysis also indicated the absence of chemical interactions between the active substance and the polymeric matrix [15].

The absence of additional bands in the spectra of the binary mixtures suggests that no new chemical bonds detectable by FTIR are formed between flunarizine and the investigated excipients. Instead, the spectra indicate a physical dispersion of the active substance within the excipient matrix, a phenomenon frequently reported in drug–excipient compatibility studies performed using FTIR.

**Figure 4:**
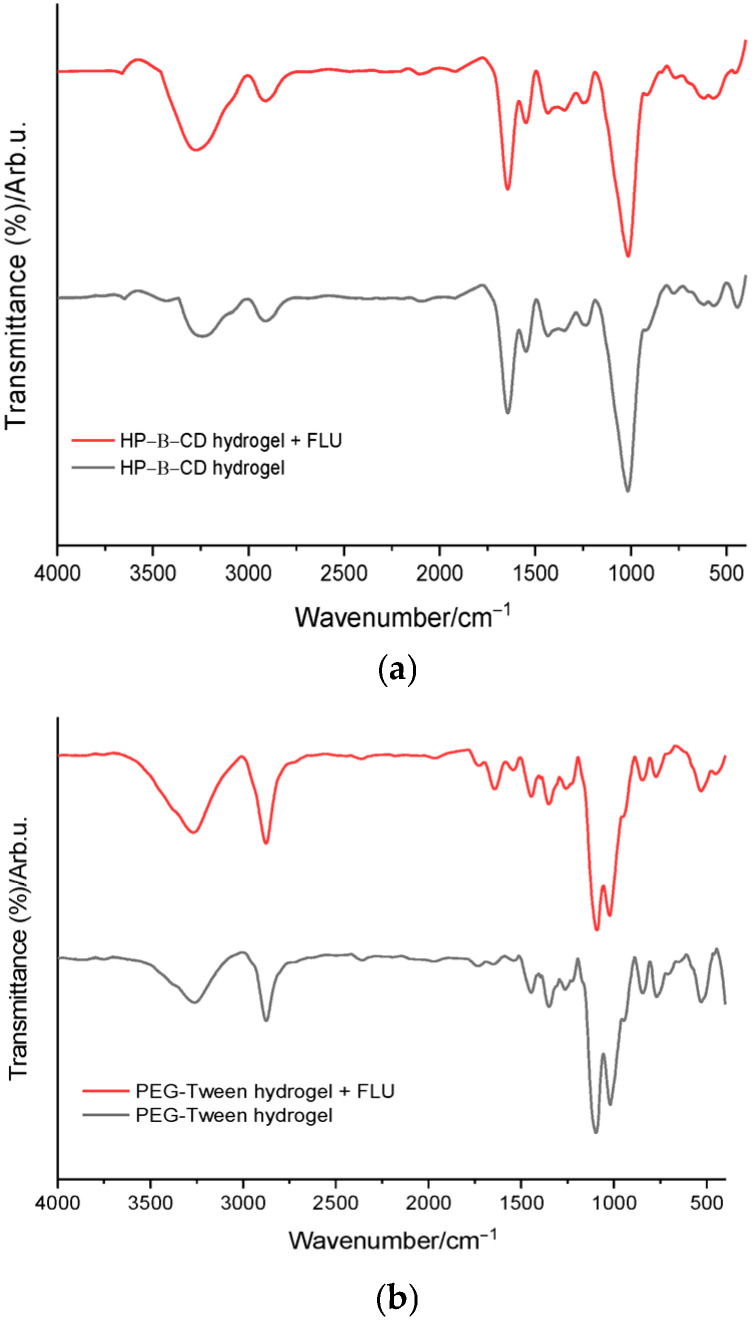
FTIR spectra of (**a**) HP-β-CD hydrogel with and without the active ingredient and (**b**) PEG-Tween hydrogel with and without the active ingredient.

The FTIR spectra of the hydrogel formulations confirm these observations. The spectra of hydrogels containing flunarizine are very similar to those of hydrogels without the active substance (Figure 4). The dominant bands are mainly associated with the polymeric structure of the excipients, particularly HP-β-cyclodextrin, PEG 6000, and Tween 20. The characteristic bands of flunarizine appear with reduced intensity or are partially overlapped by the more intense bands of the polymeric matrix. This masking effect of spectral bands is commonly observed in pharmaceutical systems in which the active substance is incorporated at relatively low concentrations within a polymeric or hydrogel matrix. The large number of hydroxyl groups and the extensive hydrogen-bonding network within the excipient matrix may dominate the spectral profile, thereby reducing the visibility of the vibrational contributions of the drug molecule [7].

A clear difference between the developed hydrogel formulations is observed when comparing systems based on HP-β-cyclodextrin with those based on PEG 6000 and Tween 20. In the case of hydrogels containing HP-β-cyclodextrin, the FTIR spectra are dominated by the broad bands associated with the hydroxyl groups of cyclodextrin, observed in the 3400–3200 cm^−1^ region, as well as by the intense bands in the 1150–1000 cm^−1^ region attributed to C–O and C–O–C vibrations of the oligosaccharide structure. In cyclodextrin systems, spectral variations may also arise from non-covalent host–guest interactions or inclusion complex formation rather than from the formation of new chemical bonds [16]. These features are typical for cyclodextrin derivatives and reflect the extensive hydrogen-bonding network within the polysaccharide matrix [7,17].

In contrast, hydrogels based on PEG 6000 and Tween 20 exhibit distinct spectral characteristics. Their spectra are dominated by the C–O–C stretching band around 1100 cm^−1^, characteristic of the polyether chains of PEG, as well as by the ester carbonyl stretching band around 1735 cm^−1^, specific to the surfactant Tween 20 [13]. In these systems, the structural contribution of the polymeric components differs from that observed in cyclodextrin-based systems, since PEG and Tween form a polymer–surfactant matrix in which the predominant interactions are hydrogen bonding and weak hydrophobic interactions.

A comparison of the spectra of hydrogels with and without flunarizine shows that the incorporation of the active substance does not lead to the appearance of new bands or major changes in the positions of the characteristic bands of the excipients in either type of system. In both cases, the characteristic bands of flunarizine are partially masked by the intense bands of the excipient matrix, a phenomenon frequently observed when drugs are incorporated into polymeric systems or hydrogel matrices [10].

However, the overall spectral profile differs between the two types of hydrogels. In systems based on HP-β-cyclodextrin, the spectrum is dominated by the polysaccharide contribution and the broad O–H band, whereas in PEG/Tween systems the vibrations of polyether chains and ester groups predominate. These differences reflect the distinct chemical nature of the two excipient matrices and indicate that the overall hydrogel structure is largely governed by the polymeric components. FTIR analysis indicates good compatibility of flunarizine with both HP-β-cyclodextrin-based systems and PEG 6000/Tween 20-based hydrogels, with no evidence of detectable chemical interactions. The differences observed between the spectra of the two types of hydrogels are mainly attributed to the different chemical nature of the excipients forming the hydrogel matrix.

In both systems, the spectra are dominated by contributions from O–H and C–O–C vibrations, reflecting the high content of hydroxyl groups and ether linkages characteristic of the polymeric excipients. In systems based on HP-β-cyclodextrin, the spectrum is mainly governed by the polysaccharide structure and the broad O–H band, whereas in PEG/Tween systems the vibrations of polyether chains and ester groups predominate.

These differences reflect the distinct chemical nature of the two excipient matrices and indicate that the overall hydrogel structure is largely governed by the polymeric components. FTIR analysis indicates good compatibility of flunarizine with both HP-β-cyclodextrin-based systems and PEG 6000/Tween 20-based hydrogels, with no evidence of detectable chemical interactions. The differences observed between the spectra of the two types of hydrogels are mainly attributed to the different chemical nature of the excipients forming the hydrogel matrix.

### 3.2. Thermal Analysis

**Figure 5:**
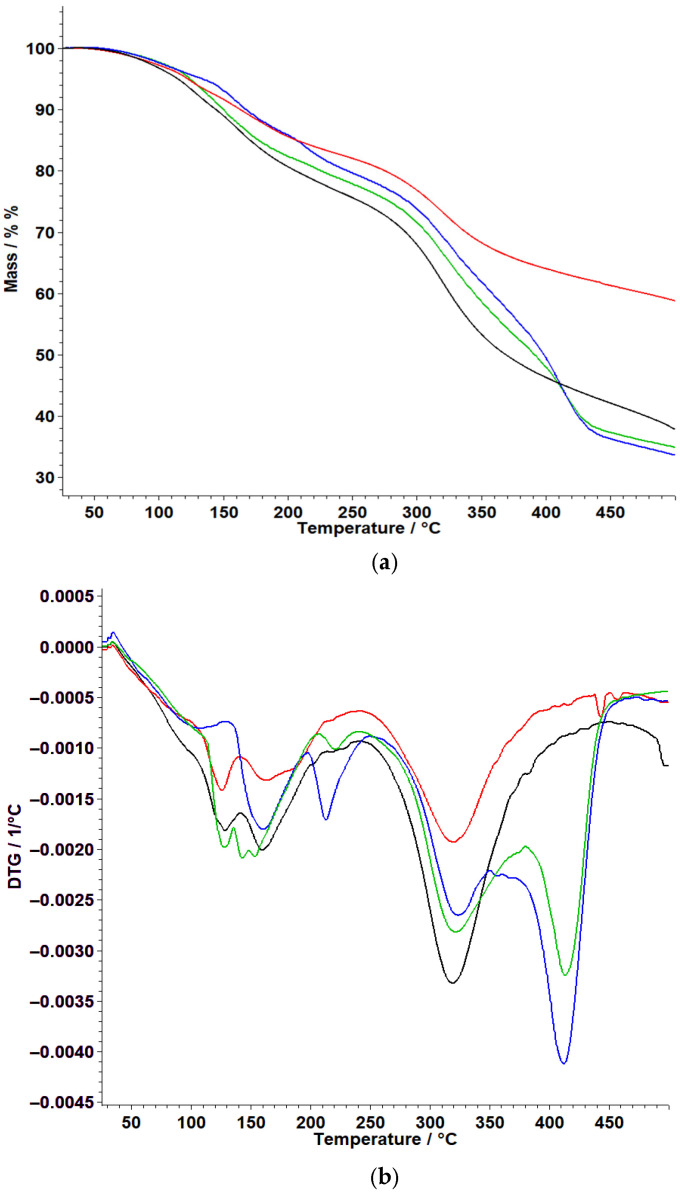
Graphical representation of TG (**a**) and DTG (**b**) curves for analyzed hydrogel sample (black—HP-β-CD hydrogel, red—HP-β-CD hydrogel + FLU, blue—PEG–Tween hydrogel, green—PEG–Tween hydrogel + FLU).

**Figure A1:**
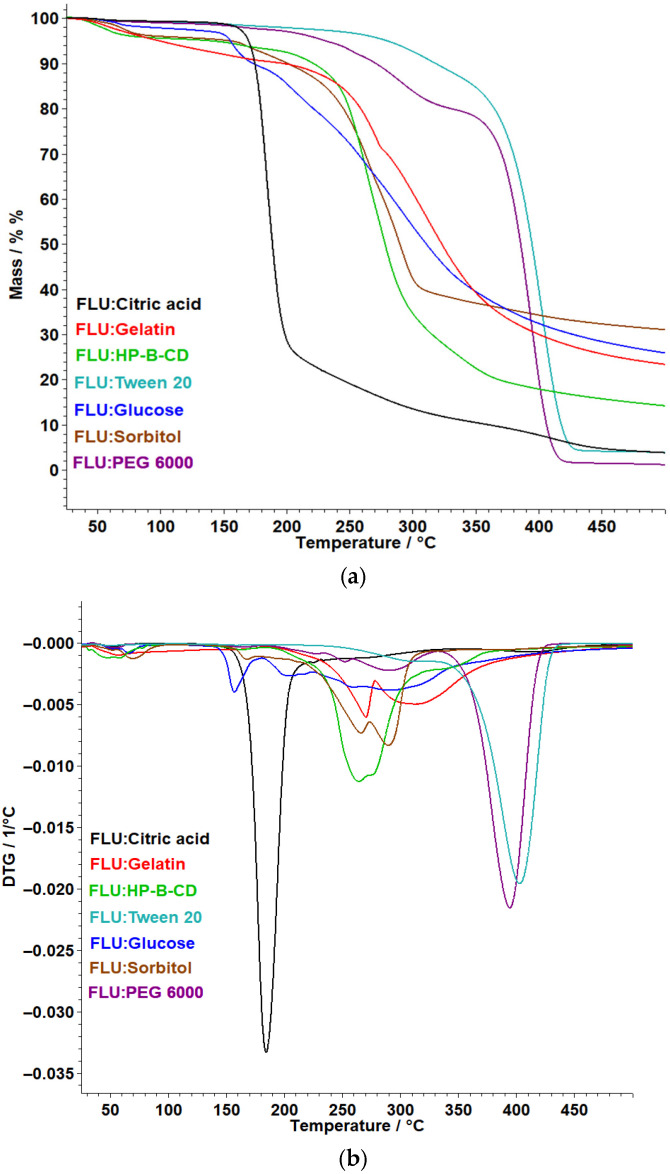
TG curves (**a**) and DTG (**b**) curves of binary mixtures 1:1 (*w*/*w*).

Thermal behavior of the binary mixtures (Figure A1a,b) and the prepared hydrogels (Figure 5) was investigated using TG/DTG analysis in order to evaluate their thermal stability and to assess potential compatibility between flunarizine and the excipients. The main thermal events and corresponding mass losses are summarized in Table 1.

A brief analysis of the individual components was first considered to support the interpretation of the hydrogel systems. The thermal behavior of the individual compounds, including the active ingredient, is illustrated in the Supplementary Materials (Figure S1a,b). Flunarizine dihydrochloride exhibits a characteristic thermal decomposition profile, which contributes to the overall thermal behavior of the binary mixtures and drug-loaded systems.

The discussion focused on the main matrix-forming excipients, which predominantly determine the thermal behavior of the hydrogel system, while the other components were not considered in detail due to their well-established thermal profiles. HP-β-cyclodextrin exhibits an initial mass loss below 100 °C, attributed to the release of adsorbed and bound water, followed by a major degradation stage associated with the decomposition of the carbohydrate framework [18,19]. In contrast, Tween 20 shows an almost complete mass loss over a broad temperature range, with several successive DTG maxima, while PEG 6000 presents a major decomposition event at higher temperatures, characteristic of polyether chain degradation [20,21,22]. This behavior is relevant for interpreting the degradation processes observed in the hydrogel systems.

The thermal parameters of the binary mixtures are summarized in Table 1 and were used to support the interpretation of the hydrogel systems. Comparison with the individual components indicates that the main degradation stages are preserved in the binary mixtures, with only minor shifts in the characteristic temperatures.

For the PEG6000/Tween20 hydrogel containing flunarizine, three degradation stages are observed. The first stage, occurring between 61 and 241 °C, shows two DTG maxima at 124 and 152 °C and a mass loss of 21.04%. This process can be associated mainly with the elimination of free and bound water retained within the hydrogel matrix, with a possible contribution from volatile components. The second stage, observed in the range 243–385 °C, with a DTG maximum at 325 °C and a mass loss of 26.42%, may be attributed predominantly to the degradation of Tween 20, with a possible contribution from the initial decomposition of PEG6000 [21,22]. The third stage, between 385 and 476 °C, with a DTG maximum at 418 °C and a mass loss of 15.87%, corresponds to the further degradation of the PEG-based organic fraction [20].

In the PEG6000/Tween20 hydrogel without flunarizine, two degradation stages are observed. The first process takes place between 61 and 243 °C, with DTG maxima at 158 and 215 °C and a mass loss of 19.47%, and can likewise be associated with water removal from the hydrated matrix. The second stage, between 244 and 466 °C, shows DTG maxima at 328 and 419 °C and a mass loss of 44.60%, indicating the main degradation of the PEG/Tween matrix [20,21,22]. Compared with the flunarizine-loaded system, the main degradation events remain in comparable temperature domains, although the distribution of mass loss between the stages differs.

The separation of degradation steps observed in the drug-loaded system may also indicate a slight modification of the thermal decomposition pathway due to the presence of the active ingredient, without suggesting the formation of new chemical entities.

For the HP-β-CD-based hydrogel containing flunarizine, two degradation stages are recorded. The first stage occurs between 49 and 244 °C, with a DTG maximum at 120 °C and a mass loss of 17.41%, and can be attributed to the release of water associated with the cyclodextrin-containing matrix [18,19]. The second stage, between 245 and 435 °C, with a DTG maximum at 329 °C and a mass loss of 20.08%, corresponds to the main degradation of the HP-β-CD-based structure [18].

A similar profile is observed for the hydrogel without flunarizine, where the first process occurs between 55 and 242 °C, with a DTG maximum at 158 °C and a mass loss of 23.36%, followed by a second degradation stage between 242 and 444 °C, with a DTG maximum at 322 °C and a mass loss of 33.57%. The presence of the same two principal degradation domains in both systems supports the preservation of the characteristic thermal behavior of HP-β-CD in the final formulations. The lower mass loss in the first stage of the drug-loaded system may reflect differences in water retention within the cyclodextrin-based matrix [19].

The thermal behavior of the hydrogels reflects the combined contribution of the excipient matrix and the incorporated drug. The observed thermal profiles allow differentiation between the main degradation domains of the excipients and those associated with the active substance, without indicating the formation of new decomposition pathways. In the present case, the hydrogel profiles can be interpreted as the result of the combined thermal behavior of the excipients and the incorporated drug, with no evidence of entirely new decomposition domains that would indicate major chemical incompatibilities [23].

Overall, the TG–DTG data indicate that flunarizine incorporation does not significantly alter the main degradation temperature domains of the investigated hydrogels. Although differences in the distribution of mass loss between the thermal stages are observed, the main thermal degradation domains remain comparable. These findings support the thermal compatibility of flunarizine with both hydrogel matrices and are consistent with the FTIR results.

### 3.3. RP-HPLC

- Chromatographic conditions

**Figure 6:**
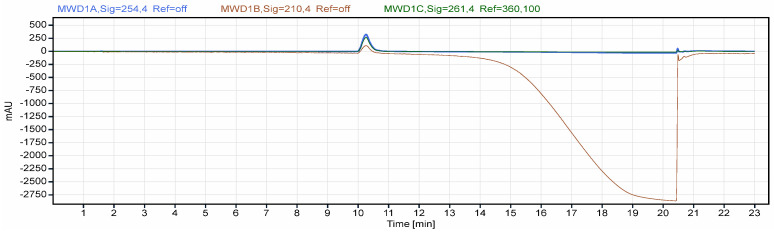
API chromatogram obtained using Waters ACQUITY APC AQ 125.

A preliminary study was performed using a Waters ACQUITY APC AQ 125 column (2.5 µm, 4.6 × 75 mm, REF 186006978); however, the system suitability conditions could not be achieved due to the secondary interactions of the target analyte with the silanol groups during the method optimization phase. Peak broadening and tailing can be seen in Figure 6. Adequate separation of the target analyte was subsequently achieved using an Agilent Eclipse XDB-C18 column (5 µm, 4.6 × 150 mm, PN 993967-902).

The aqueous mobile phase consisted of a 45 mM solution of ammonium formate in ultrapure water, acidified with 0.4% (*v*/*v*) formic acid, and the organic phase selected was gradient grade acetonitrile. The column was conditioned by increasing the concentration of the aqueous mobile phase with 10%/min until reaching the initial method conditions (95% aqueous, 5% organic), followed by the column equilibration time of one hour prior to injecting samples. An injection volume of 5 µL was used to allow for good repeatability and minimize the column loading. Column temperature was maintained at 40 °C during the run. The final gradient conditions were selected after multiple injections of API and excipients to avoid interferences.

Chromatograms were recorded at 210, 254 and 261 nm, using a 4 nm bandwidth with no reference wavelength. The maximum sensitivity was obtained at the 254 nm wavelength, measuring an S/N ratio of 82.4 for 5 ng of flunarizine on column. The 210 nm wavelength was utilized for impurity profiling, as most organic substances exhibit absorbance in that UV range, allowing for the detection of non-aromatic impurities. The 254 nm wavelength, corresponding to the maximum absorbance of flunarizine, was selected for the final method due to its increased sensitivity. Additionally, 261 nm was evaluated for selectivity; while it offered a more stable baseline and reduced susceptibility to background noise, it provided lower sensitivity compared to 254 nm.

**Figure 7:**
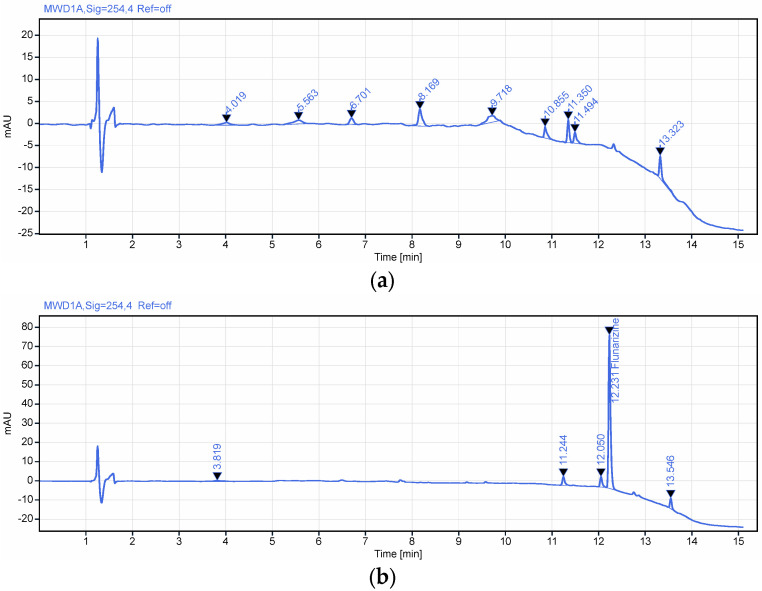
Representative chromatograms for (**a**) excipients without API (gradient 1) and (**b**) excipients spiked with API (gradient 1). RT for flunarizine 12.231 min.

**Figure 8:**
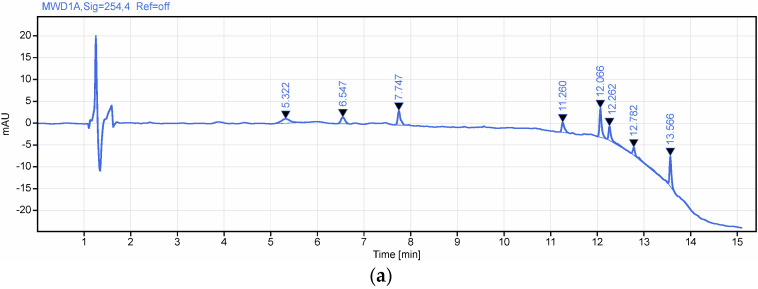

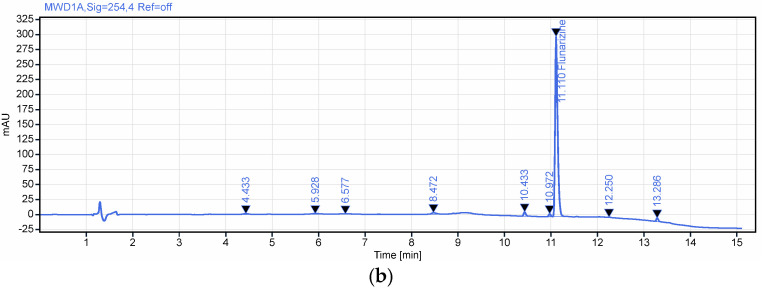
Representative chromatograms for (**a**) excipients without API and (**b**) excipients spiked with API (gradient 2). RT for flunarizine 11.110 min.

Two gradient programs were evaluated during method development to assess selectivity and optimize chromatographic performance. For gradient 1 (Table 2), no significant interferences were observed (Figure 7), demonstrating adequate selectivity for the analyte of interest. However, the total run time (25 min) was considered too long. For gradient 2 (Table 3), an increased gradient slope was applied, which did not result in the appearance of any additional interferences (Figure 8), thereby maintaining method selectivity. This modification led to a reduction in analysis time to 22.1 min, decreasing mobile phase consumption and enhancing sample throughput.

- Method validation

The analytical method developed for the assay of flunarizine in hydrogel form requires validation to demonstrate that it is selective, sensitive, and reproducible since the excipients used in this novel formulation could interfere with the chromatographic separation. A consolidated validation approach was adopted to evaluate both formulations simultaneously. This strategy was chosen because the hydrogel matrices share a common foundational composition. By including both PEG 6000 and hydroxypropyl-β-cyclodextrin variants in a single validation suite, we successfully demonstrated this method’s robustness and selectivity against potential excipient interferences.

System suitability

The system suitability test is useful in determining if the chromatographic system is suitable for the required analysis by repeatedly injecting a standard solution and analyzing the chromatographic parameters of the target analyte peak. This test is mandatory for all RP-HPLC applications, as specified in the EP and USP [24]. The system suitability results (Table 4) demonstrate that the chromatographic method meets the acceptance criteria. The percentage relative standard deviation (%RSD) for peak area (0.72%) and retention time (0.25%) indicates excellent repeatability of the system. The average tailing factor (1.699) falls within acceptable limits, confirming appropriate peak symmetry. Furthermore, the capacity factor (k′ ≈ 9.07) is within the optimal range, indicating adequate retention of the analyte. The high number of theoretical plates (N ≈ 126,851) reflects excellent column efficiency. Collectively, these parameters confirm that the chromatographic system is suitable for the intended analytical application.

Linearity

**Figure 9:**
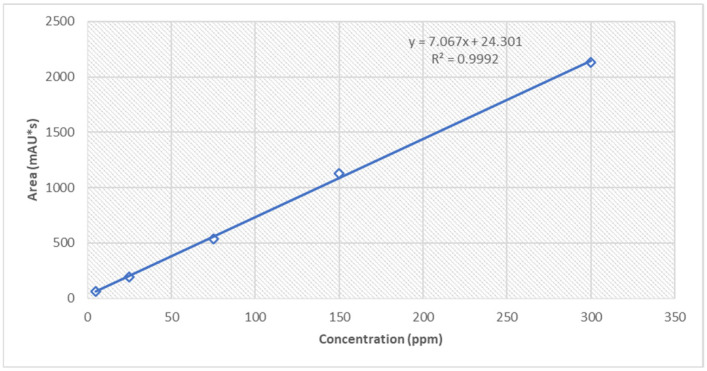
Standard calibration curve for flunarizine.

The calibration curve (Figure 9) for the API was obtained by repeatedly injecting 5 µL of diluted standard solution for each level at 5, 25, 75, 150 and 300 µg/mL, plotting the measured peak area (Table 5) against each concentration level. The relationship between peak area and concentration is linear within the calibration range described above [24].

Intermediate Precision

The intermediate precision of the method was evaluated by analyzing samples at three concentration levels (120, 150, and 180 µg/mL) over two different days (n = 3 at each level, per day). This design ensures that the analytical method for flunarizine is robust against daily variations in mobile phase preparation and environmental conditions across the intended linear range. The results (Table 6), expressed as the percentage relative standard deviation (%RSD) of the recovery values, were found to be well within the acceptable ICH limit of <2.0%, confirming the method’s high degree of reproducibility and reliability [25].

Accuracy

A concentrated spiking solution was prepared by accurately weighing 10.3 mg of flunarizine dihydrochloride into a 5 mL volumetric flask and diluting to volume with methanol (final concentration 2.06 mg/mL). To simulate the 80%, 100%, and 120% target levels, aliquots of 300, 375, and 450 µL of this stock solution were transferred into placebo hydrogel matrices and extracted with methanol, according to the sample preparation procedure. Recovery was calculated using Ecquations (1)–(4) (see Table A1 for nomenclature). The acceptance criteria for recovery are 100 ± 2% and the RSD value measured for each replicate set must be lower than 2% [25]. The results indicate that the method is accurate across the tested concentration range, as the recoveries fall within commonly accepted limits (Table 7).

(1) $$S_{F}=\frac{W_{SS}}{W_{NS}}\;$$

(2) $$F_{S}=\frac{W_{M}}{W_{N}}\;$$

(3) $$S_{A}=S_{c}\times S_{V}$$

(4) $$Recovery\;\left(\%\right)=\frac{M_{A}\times F_{S}}{S_{A}\times S_{F}}\times 100\;$$

Sensitivity

The sensitivity of the analytical procedure for flunarizine was evaluated using the Signal-to-Noise (S/N) ratio. The limit of detection (LOD) was established at 0.05 µg/mL (S/N 3) and the limit of quantitation (LOQ) was established at 0.16 µg/mL (S/N 10). These results demonstrate that the method is sensitive enough to monitor flunarizine at concentrations significantly below the nominal concentration range [26].

Specificity and selectivity

**Figure 10:**
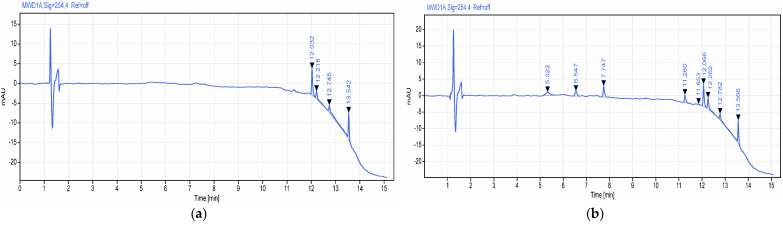

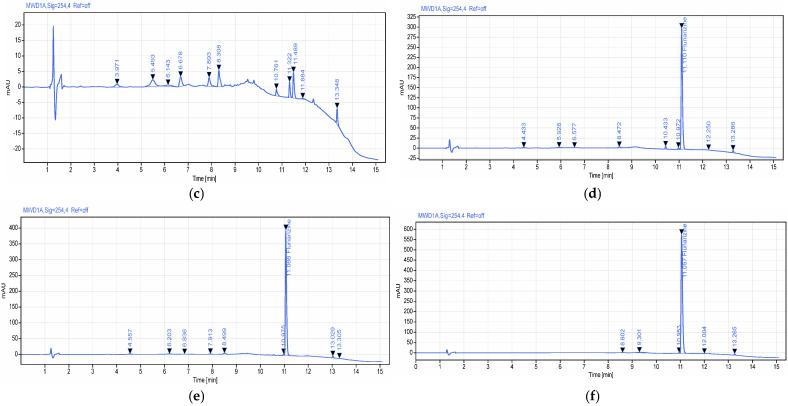
Specificity and selectivity test chromatograms for: (**a**) solvent blank chromatogram; (**b**) formulation no. 1 matrix blank chromatogram; (**c**) formulation no. 2 matrix blank chromatogram; (**d**) formulation no. 1 spiked placebo chromatogram; (**e**) formulation no. 2 spiked placebo chromatogram; and (**f**) standard API chromatogram.

The RP-HPLC method for the assay of flunarizine in the hydrogel formulation was evaluated for specificity and selectivity to ensure reliable measurement of the active ingredient in the presence of excipients. Specificity was confirmed by analyzing blank solvent, placebo matrix, and the reference standard, demonstrating no interfering peaks at the retention time of flunarizine (Figure 10). Selectivity was assessed on a matrix-spiked sample by verifying the resolution between flunarizine and the closest eluting peak at 10.97 min, determining a resolution factor (EP) of 1.51, ensuring baseline separation. These results demonstrate that the method is both specific and selective for quantifying flunarizine in the hydrogel dosage form [24,25].

Robustness-Gradient changes

The robustness of the gradient profile was evaluated by varying the slope of the organic phase (Phase B). To ensure method compatibility across various HPLC systems with differing dwell volumes, robustness was tested using a shallower slope of 9% B/min (Table 8, B). This variation also simulates potential inaccuracies in pump proportioning and solvent delivery. The method is considered robust as all system suitability criteria (Table 9 and Table 10), specifically the resolution factor, remained above 1.5 across both conditions [27].

- Sample results

The assay results obtained using Equations (2), (5) and (6) (see Table A1 for nomenclature) for both hydrogel formulations are in close agreement with the theoretical concentrations (Table 11). Furthermore, the measured API content for all batches fell within the ±10% tolerance range of the label claim, satisfying the standard acceptance criteria for pharmaceutical dosage forms. The absence of detectable peaks above the limit of quantification (LOQ) in both blank formulations confirms the specificity of the analytical method and demonstrates that no interference arises from the excipient matrix. Collectively, these findings support the reliability of the method for the quantification of flunarizine in the developed hydrogel systems.

(5) $$C_{S}=\frac{{Sample}_{area}-Intercept}{Slope}$$

(6) $$Assay\;\left(mg/g\right)=\frac{C_{s}\times V_{d}\times F_{S}\times P}{S_{w}}\;$$

## 4. Conclusions

In this study, hydrogel-based formulations containing flunarizine dihydrochloride were developed and characterized using complementary physicochemical and analytical techniques.

FTIR spectroscopy demonstrated that the spectra of the binary mixtures and hydrogel formulations correspond mainly to the superposition of the characteristic absorption bands of the individual components, without the appearance of new bands or significant shifts in the main vibrational regions. These observations indicate no evidence of detectable chemical interactions between flunarizine and the selected excipients and suggest that the drug is incorporated into the matrices mainly through physical dispersion and weak intermolecular interactions.

Thermal analysis (TG/DTG) supports these findings, as the thermal decomposition profiles of the mixtures and final formulations preserve the characteristic degradation stages of the individual components. For the PEG–Tween hydrogel containing flunarizine, three degradation stages were observed with mass losses of 21.04% (61–241 °C; DTGmax 124 and 152 °C), 26.42% (243–385 °C; DTGmax 325 °C), and 15.87% (385–476 °C; DTGmax 418 °C). The HP-β-CD hydrogel containing flunarizine exhibited two stages with mass losses of 17.41% (49–244 °C; DTGmax 120 °C) and 20.08% (245–435 °C; DTGmax 329 °C). The absence of additional decomposition processes indicates that no new chemical species are formed during the formulation process and that flunarizine remains thermally stable within the hydrogel systems.

The validated RP-HPLC method demonstrated high specificity, linearity, and precision for the quantification of flunarizine in the developed hydrogel systems. The method was linear over 5–300 µg/mL (R^2^ = 0.9992) and sensitive (LOD 0.05 µg/mL; LOQ 0.16 µg/mL). The measured drug content in the formulations was 1.87 mg/g (95.50% label claim) for the PEG–Tween hydrogel + FLU and 1.93 mg/g (97.86% label claim) for the HP-β-CD hydrogel + FLU, confirming reliable assay performance and lack of interference from the excipient matrices.

The consistency of the results obtained by FTIR, TG/DTG, and HPLC provides converging evidence that flunarizine dihydrochloride is compatible with the selected excipients and can be successfully incorporated into both cyclodextrin-based and PEG/Tween hydrogels with preserved spectral profiles, maintained thermal degradation domains (DTGmax values in comparable ranges), and assay values within the usual ±10% acceptance range for label claim (95.50–97.86%). These findings highlight the potential of these formulations as alternative pharmaceutical delivery systems and provide a solid basis for further development and investigation.

## Figures and Tables

**Table 1:** Characteristic thermal parameters (temperature ranges, DTG_max_ and mass loss) obtained from TG/DTG analysis of binary mixtures and hydrogel systems.

Sample	Process	TG		DTG_max_/°C	Δm/%
		T_onset_/°C	T_final_/°C		
FLU:Citric acid	I	158	223	184	75.20
FLU:Gelatin	I	41	103	59	5.05
	II	173	282	274	20.80
	III	282	462	315	44.89
FLU:HP-β-CD	I	41	99	55	4.03
	II	132	383	260	76.05
FLU:Tween 20	I	234	468	405	93.25
FLU:Glucose	I	51	94	68	1.92
	II	117	477	156	70.43
FLU:Sorbitol	I	43	100	69	3.84
	II	144	171	168	1.70
	III	217	377	270, 293	52.17
FLU:PEG 6000	I	171	484	398	96.49
PEG–Tween hydrogel + FLU	I	61	241	124,152	21.04
	II	243	385	325	26.42
	III	385	476	418	15.87
PEG–Tween hydrogel	I	61	243	158,215	19.47
	II	244	466	328, 419	44.60
HP-β-CD hydrogel + FLU	I	49	244	120	17.41
	II	245	435	329	20.08
HP-β-CD hydrogel	I	55	242	158	23.36
	II	242	444	322	33.57

**Table 2:** Initial gradient program (gradient 1) at flow 0.70 mL/min.

Time (min)	%A	%B
0	98.0	2.0
3.00	98.0	2.0
7.00	85.0	15.0
15.50	0.0	100
17.00	0.0	100
18.00	98.0	2.0
23.00	98.0	2.0
Post time: 2 min		

**Table 3:** Final gradient program (gradient 2) at flow 1.20 (mL/min).

Time (min)	%A	%B
0	95.0	5.0
3.00	95.0	5.0
5.00	90	10.0
10.00	40	60.0
12.00	0.0	100.0
15.00	0.0	100.0
15.10	95.0	5.0
Post time: 7 min		

**Table 4:** System suitability parameters.

Injection	Area of Target Peak (mAU∗s)	Retention Time (min)	Tailing Factor	Capacity Factor (k′)	EP Plates (N)
1	3174.62	11.076	1.667	9.069	129,271
2	3218.61	11.094	1.670	9.085	125,935
3	3221.10	11.048	1.751	9.042	124,637
4	3220.92	11.051	1.735	9.045	123,516
5	3246.03	11.124	1.672	9.113	130,898
Average	3216.26	11.079	1.699	9.071	126,851
%RSD	0.72	0.25			

**Table 5:** Linearity data.

Concentration (µg/mL)	Area (mAU∗s)	Calc. Concentration (µg/mL)	Slope	Intercept	Corr. Coef.
5	59.151	4.931	7.067	24.301	0.9992
25	190.97	23.584			
75	540.255	73.009			
150	1124.772	155.720			
300	2128.568	297.759			

**Table 6:** Intermediate precision data.

Analyte	Concentrations (µg/mL)	Intraday Precision 1st Day	Interday Precision 2nd Day
		%RSD (n = 3)	%RSD (n = 3)
Flunarizine	120	0.16	0.20
	150	0.43	0.60
	180	0.16	0.33

**Table 7:** Accuracy and recovery data.

Level %	Spiked Amount (mg)	Average Area (n = 3) (mAU∗s)	Measured Amount (mg)	%RSD (n = 3)	Recovery %
80	0.618	907.891	0.623	0.45	100.75
100	0.773	1110.208	0.765	0.32	99.06
120	0.927	1346.864	0.932	0.17	100.54

**Table 8:** For (A) method gradient and (B) test gradient.

**(A) Method Gradient**		
**Time (min)**	**%A**	**%B**
0	95.0	5.0
3.00	95.0	5.0
5.00	90	10.0
10.00	40	60.0
12.00	0.0	100.0
15.00	0.0	100.0
15.10	95.0	5.0
Post time: **7 min**		
**(B) Test Gradient**		
**Time (min)**	**%A**	**%B**
0	95.0	5.0
3.00	95.0	5.0
5.00	90	10.0
10.00	40	55.0
12.00	0.0	100.0
15.00	0.0	100.0
15.10	95.0	5.0
Post time: **7 min**		

**Table 9:** Gradient testing results.

Parameter	Variation	Resolution	Retention Time (min)	EP Plates (N)	Tailing Factor
Gradient Slope 1 (5–10 min)	10% B/min	1.51	11.05	123,516.48	1.67
	9% B/min	1.79	11.14	225,246.52	1.32

**Table 10:** Acceptance criteria.

Parameter	Acceptance Criteria
Resolution	>1.5
Retention Time	10.83–11.27
EP Plates (N)	>100,000
Tailing Factor	<2
Capacity factor (k′)	2.0 < k′ < 10.0

**Table 11:** Sample results.

Sample Number	Sample Name	Sample Mass (mg)	Average (n = 2) Area (mAU∗s)	Assay (mg/g)	Expected Concentration (mg/g)	Label Claim (%)
1	PEG–Tween hydrogel + FLU	424.96	1159.86	1.87	1.96	95.50
2	PEG–Tween hydrogel	472.52	N/A	Below LOQ	N/A	N/A
3	HP-β-CD hydrogel + FLU	81.93	249.79	1.93	1.97	97.86
4	HP-β-CD hydrogel	85.87	N/A	Below LOQ	N/A	N/A

## Data Availability

The data presented in this study are available on request from the corresponding authors.

## Supplementary Materials

The following supporting information can be downloaded at: https://www.mdpi.com/article/10.3390/polym18091014/s1, Figure S1: TG curves (a) and DTG curves (b) of flunarizine dihydrochloride and the individual components of the hydrogels.

## Appendix A

**Table A1:** Nomenclature and units for the analytical assay calculation.

Variable	Description	Unit
*S_F_*	Spike solution factor	Dimensionless
*W_SS_*	Weighed standard for spike solution preparation	mg
*W_NS_*	Nominal standard mass for spike solution preparation	mg
*F_S_*	Standard solution factor	Dimensionless
*W_M_*	Weighed standard amount	mg
*W_N_*	Nominal standard mass (5 mg)	mg
*S_A_*	Spiked amount	mg
*S_C_*	Spike solution concentration	mg/mL
*S_V_*	Added spike solution volume	mL
*C_S_*	is the measured flunarizine concentration	mg/mL
*V_d_*	is the extraction solvent volume used for dissolution	mL
*P*	Potency of the flunarizine standard (value from CoA)	%
*S_w_*	Sample weight	g
