# Supplementary material for: Flunarizine-Loaded Hydrogels: A Novel Formulation and Physicochemical Characterization
Source: Polymers (Basel). 2026 Apr 22;18(9):1014. doi: 10.3390/polym18091014 (PMC13164590; doi:10.3390/polym18091014)
Supplement: Supplementary file 1 [file polymers-18-01014-s001.zip › polymers-4259484-supplementary.pdf]

Supplementary Materials

Flunarizine-Loaded Hydrogels: A Novel Formulation and Physicochemical Characterization

Camelia Daniela Ionaș [1,2], Dorinel Okolišan [1,2], Camelia Epuran [1], Ion Frățilescu [1], Gabriela Vlase [1,2*], Alexandru Pahomi [1], Raul Ștefan-Pantiș [1,2], Mihaela Maria Budiul [1*], Mădălina Grădinaru [1] and Titus Vlase [1,2,]

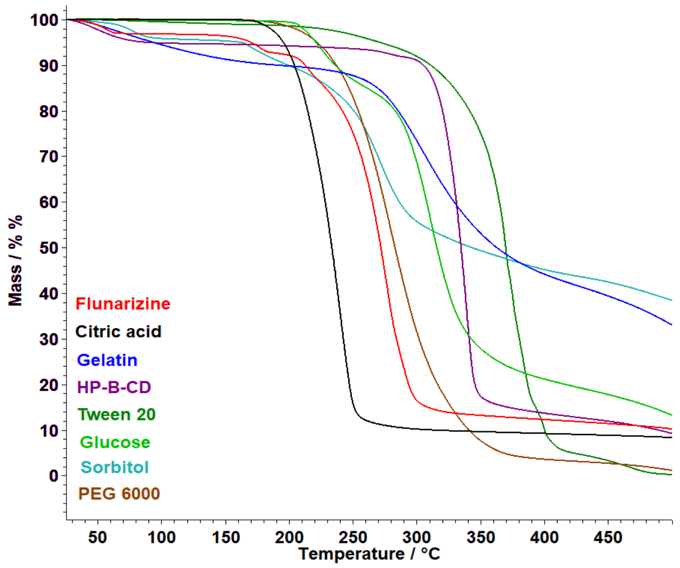

(a)

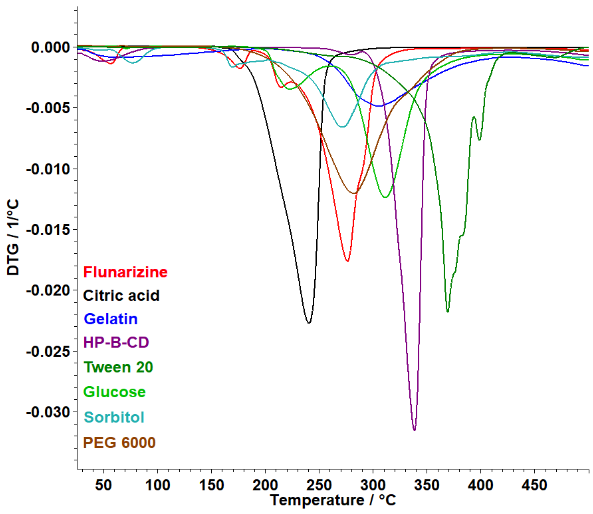

(b)

Figure S1. TG curves **(a)** and DTG curves **(b)** of flunarizine dihydrochloride and the individual components of the hydrogels
